# Supplementary material for: Effect of Defatting Method on Japanese Quince (Chaenomeles japonica) Fruit Seed Protein Isolate Technological Properties
Source: Foods. 2025 Jan 13;14(2):234. doi: 10.3390/foods14020234 (PMC11765201; doi:10.3390/foods14020234)
Supplement: Supplementary file 1 [file foods-14-00234-s001.zip › foods-3399535-supplementary.pdf]

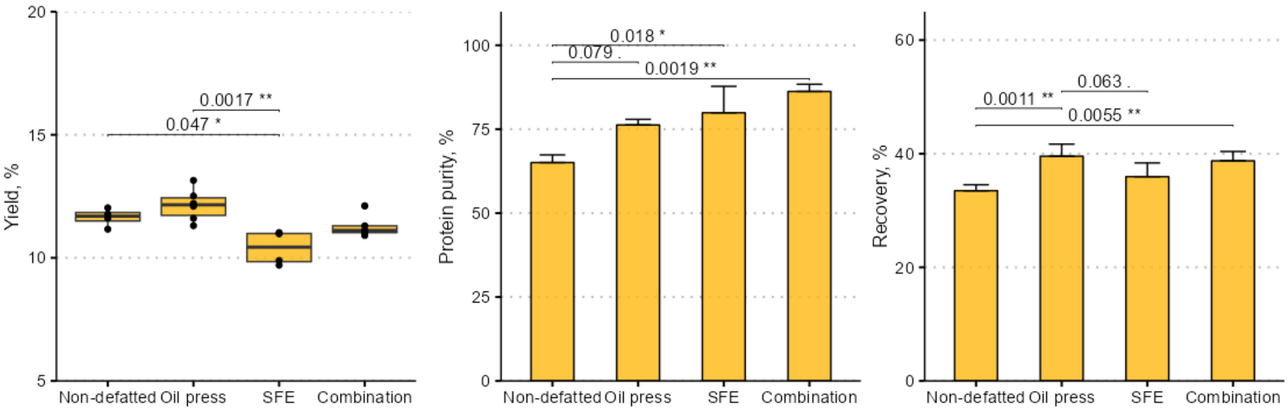

(a) **(b)** **(c)**

Figure S1. (a) Protein yield, **(b)** purity (protein content) and **(c)** recovery depending on defatting method. Statistically significant differences between samples with *p*-values followed by significance indicators (., p<0.1; *, p<0.05; **, p<0.01) are provided above boxplots.

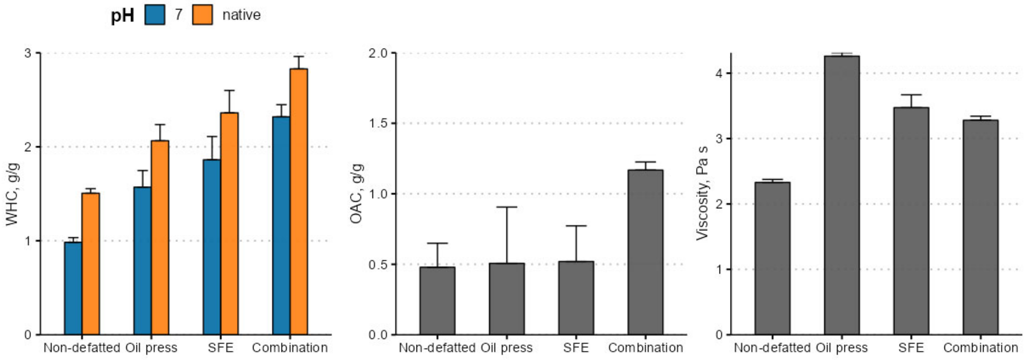

(a) **(b)** **(c)**

Figure S2. Japanese quince seed protien extract **(a)** water holding capacity, g g^{-1}; **(b)** oil binding capacity, g g^{-1}; and (c) apparent viscosity, Pa s. Data are presented as means + standard deviation of three replications (n=3).

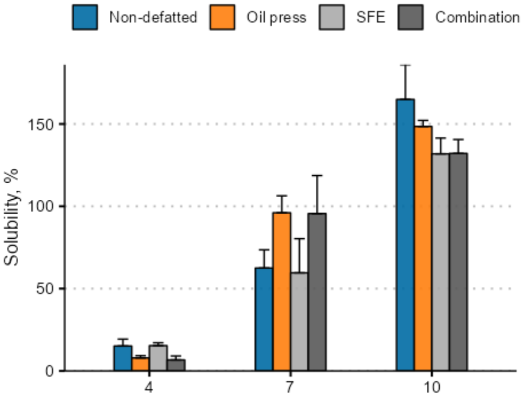

(a)

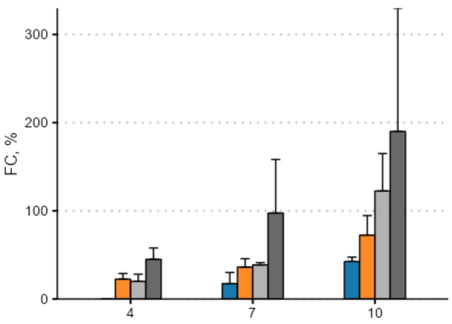

(b)

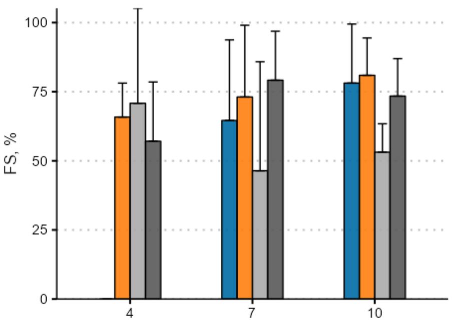

(c)

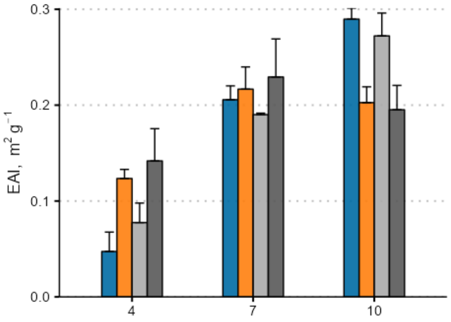

(d)

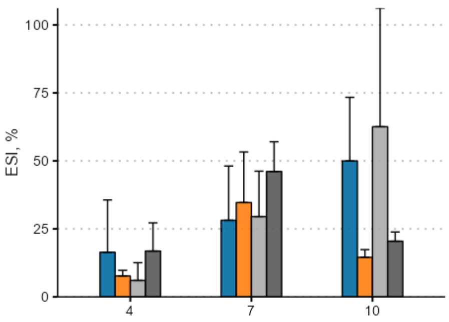

(e)

Figure S3. Functional properties of Japanese quince seed protein across pH: **(a)** Protein solubility, % of theoretically dissolvable protein; **(b)** Foaming capacity, % of initial volume; **(c)** foam stability, % of initial volume; **(d)** emulsifying activity index, m^2 g^{-1}; **(e)** emulsion stability index, % of initial EAI after 30 minutes. Data are presented as means + standard deviation of two replications from two extraction batches, each (n=4).

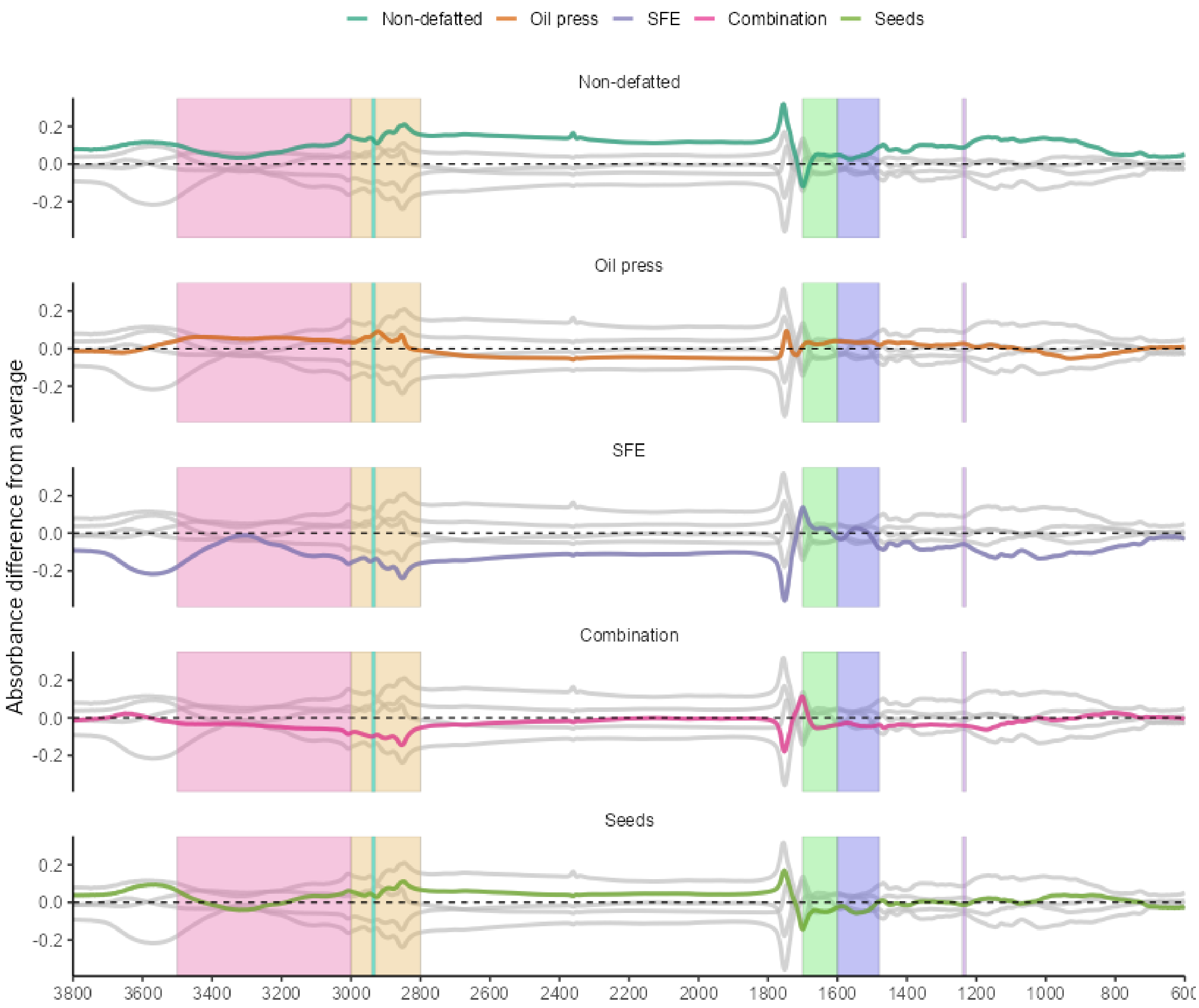

Figure S4. Difference between mean and average absorbance across the DR-FTIR spectrum. For easier comparison, relevant ranges in the FTIR spectrum are provided as colored bands: amide I (green, 1600 – 1700 cm^{-1}), amide II (blue, 1480 – 1600 cm^{-1}), amide III (purple, 1230 – 1240 cm^{-1}), C-H stretch (orange, 2800 – 3000 cm^{-1}), amide A (pink, 2000 – 3500 cm^{-1}) and amide B (cyan, 2930 – 2940 cm^{-1}).
